# Experiences and perceptions of youth living with HIV in Western Uganda on school attendance: barriers and facilitators

**DOI:** 10.1186/s12889-020-8198-7

**Published:** 2020-01-17

**Authors:** Emmanuel Kimera, Sofie Vindevogel, Mugenyi Justuce Kintu, John Rubaihayo, Jessica De Maeyer, Didier Reynaert, Anne-Mie Engelen, Fred Nuwaha, Johan Bilsen

**Affiliations:** 1 Department of Public Health, School of Health Sciences, Mountain of the Moon University, Fort Portal, Uganda; 2 Department of Orthopedagogy, Faculty of Education, Health and Social Work, University of Applied Sciences and Arts Gent, Ghent, Belgium; 3 Department of Public Health, Mental Health and Wellbeing research group, Vrije Universiteit Brussels, Brussels, Belgium; 4 Department of Occupational therapy, Faculty of Education, Health and Social Work, University of Applied Sciences and Arts Gent, Ghent, Belgium; 5 College of Health Sciences, School of Public Health, Makerere University, Kampala, Uganda

**Keywords:** HIV, Youth, School, Qualitative, Barriers, Challenges, Facilitators, Stigma, Disclosure

## Abstract

**Background:**

The globally recognized socio-economic benefits of education have stirred many countries in Sub-Saharan Africa like Uganda to promote universal access to schooling by removing fiscal barricades for those in primary and secondary schools. However, the proportion of Youth Living With HIV/AIDS (YLWHA) missing school, studying with difficulties and dropping out of school in Uganda has been observed to be higher than that of other youth. This study aimed at understanding the barriers and facilitators for YLWHA in Uganda to attend school.

**Methods:**

We conducted a qualitative inquiry with 35 purposively selected YLWHA aged 12 to 19 years, including 16 females at three accredited Antiretroviral Therapy (ART) treatment centres in Kabarole district in Western Uganda. Individual semi-structured interviews were tape-recorded, transcribed verbatim and subjected to thematic inductive analysis.

**Results:**

We identified five main themes in which barriers to attend school were reported and four main themes in which facilitators were reported by participants. The main themes for barriers were: 1) management of ART and illnesses, 2) fear, negative thoughts and self-devaluation, 3) lack of meaningful and supportive relationships, 4) reactionary attitudes and behaviours from others at school, 5) financial challenges. The main themes for facilitators were: 1) practical support at school, home and community, 2) counselling, encouragement and spirituality, 3) individual coping strategies, 4) hopes, dreams and opportunities for the future.

**Conclusion:**

Most of the barriers reported arose from HIV-related stigma and financial challenges whose genesis transcends school boundaries. While YLWHA reported measures to cope, and support from other people, these were non-sustainable and on a limited scale due to disclosure apprehension at school and the indiscretion of those who learnt about their status. To promote supportive school environments for YLWHA, integrated curricular and extracurricular interventions are necessary to increase HIV knowledge, dispel misconceptions about HIV and consequently transform the school community from a stigmatizing one to a supportive one.

## Background

While perinatal HIV transmission has been substantially reduced in the past decade, an estimated 1.8 million young people live with HIV globally with 30% of all new infections occurring among youth aged 15–24 years [1]. Uganda currently counts approximately 170,000 Youth Living With HIV/AID (YLWHA) and figures are expected to rise as more youth remain highly vulnerable to the infection [2] and as access to Antiretroviral Therapy (ART) increases [3–5]. This group’s increased life expectancy urges for a better understanding of their psychosocial situation and Quality of Life (QoL) [6]. Quality of life is a framework used in supporting people living with a chronic condition since it focuses on the general wellbeing and satisfaction with life in different life domains that may be affected by the condition [7]. Since youth spend most of their time in school [6] and since education has been widely promoted as a sustainable development goal [8] and a human right [9], it is important to explore barriers and facilitators to attend school for those infected with HIV. Improving the conditions at school can positively impact on the attendance and QoL of those living with chronic illnesses such as HIV/AIDS. As proposed by Gauri, YLWHA should have equal access to education, treatment and care for their special needs to enhance their physical, emotional, social and personal development [10].

It is widely recognized that health and education are intertwined, and schools are ideal places to strive and attain both [11]. Schools constitute an important developmental context for youth and shape their social, emotional, cognitive and behavioral functioning [12]. Additionally, at school, youth develop skills and values needed later in life [13]. Also, Smith and colleagues proposed that schools can act as centers for comprehensive HIV/AIDS response since they are ideal places to bring together teachers, caregivers and others to care for vulnerable youth [14]. Moreover, in Sub-Saharan Africa (SSA) where HIV has disrupted the social function of family, schools are being viewed as potential substitutes [15]. However, decreased school attendance has been reported as one of the impacts of HIV infection in children [16]. While in resource-limited countries like Uganda all youth experience structural and individual barriers to schooling, their HIV-positive serostatus makes this even worse, and is significantly different from other chronic conditions [17]. Living with HIV in the school community is an exceptionally vexing problem since one not only has to cope with the challenges of the disease as such, but also with the anticipated and overt reactions from their environment [18, 19]. However, until now the schooling challenges for YLWHA have attracted little attention [20]. Besides the direct detrimental effects on these youth and their environment, this negligence in addressing schooling concerns of such a large number of YLWHA, has adverse socio-economic effects in countries of SSA like Uganda with youthful populations [21] in the long run e.g. contributing to a lack of sufficient skilled work force and leadership in the future [22, 23].

Prior studies in other countries have elaborated some barriers and facilitators to attend school for YLWHA. Significant barriers include HIV-related stigma e.g., [6, 24], financial deficiencies and poor health [18]. Additionally, Baxen and Haipinge reported inadequate support in schools for YLWHA due to non-disclosure of HIV status [25]. Inadvertent disclosure that usually occurs as a result of lack of privacy for HIV-positive leaners to take medication at school, frequent illnesses, and frequent visits to the ART clinics [26], often led to discrimination of YLWHA and in some cases dropping out of school [27]. Teachers were also reported to be indiscreet, ignorant about HIV/AIDS, non-caring and non-responsive to taunts from other children reported to them by students living with HIV [25].

To facilitate school attendance for HIV-positive youth, a study conducted in Swaziland [28] found that teachers promoted non-discrimination in class, provided materials like food and clothes to children in addition to extra hours to support HIV-positive children in class. The National HIV/AIDS policy for Uganda also provides for school attendance, nondiscrimination and support of all vulnerable children in school including those with HIV/AIDS. Moreover, primary and secondary education in Uganda is free due to Universal Primary Education (UPE) and Universal Secondary Education (USE) programs but what constrains or facilitates YLWHA to attend school in the Ugandan context remains unclear; aspects that this paper seeks to highlight. Such knowledge would be the basis for appropriate policies and interventions to promote school attendance for YLWHA by addressing barriers at school while enhancing and promoting the facilitators. Our study aims to answer the question: what are the perceived or experienced facilitators and barriers for YLWHA to attend school in Ugandan?

## Methods

### Study design, settings and participants

The current study was nested in a larger research project with a goal of developing a sustainable intervention to promote the QoL of YLWHA in schools and larger communities of Kabarole district in Western Uganda. This district has one of the highest numbers of YLWHA in care compared to other districts in Uganda. We used a qualitative design employing semi-structured interviews conducted between July 2018 and October 2018 with a purposively selected sample of 35 YLWHA aged 12–19 years including 16 females. We selected this age bracket to target youth in secondary schools (our target sites for interventions) and because full disclosure to those perinatally infected is expected by at least 12 years [29]. Interview guidelines focused on: general experiences of living with HIV/AIDS, disclosure of HIV status, perceived resources and barriers for living with HIV/AIDS in school communities, and what constitutes supportive school communities (see Additional file 3 for the complete interview guide that was developed for this study). The interviews were conducted in three purposively selected ART accredited Health Facilities (HF) in Kabarole district. Selection of the HFs was done in a way that ensured wider geographical coverage of the district with one HF selected in the urban, peri-urban and rural settings in order to study experiences across these three different settings. Health facilities with large numbers of HIV-positive youth in care based on records from the District Health Office (DHO) were selected to ensure a large pool of participants with varied characteristics of age, sex and school attendance (Additional file 1: Table S1). We selected a total of 35 YLWHA, using the inclusion criteria of: being aware of their HIV status (to prevent inadvertent disclosure), having been on ART for at least 6 months, being able to communicate in Rutooro or Luganda or English, being in the right mental state to participate in an interview as judged by health workers, and willingness to participate in an audio recorded interview. We intended to exclude potential participants who had other chronic conditions such as cancer as these would confound experiences of living with HIV, those with active tuberculosis disease to mitigate the risk of contagion and those whose parents (in case of minors) or themselves did not consent/assent.

### Data collection

At each HF, the ART clinic in-charge assigned a health worker to oversee the participant recruitment and data collection process. As eligible participants in the ART clinic register turned up for their clinic appointment, we purposively selected those we involved in the study and interviewed them after their clinic appointment. In a private quiet room at the HF, we provided information about the research to each potential participant after which we sought their informed consent/assent. We initially planned to seek consent from parents/caretakers of minors at the HF, but we realized that these youth were never accompanied by their parents/caretakers. For this category, parental/caretaker consent was sought in advance of their visit through phone calls inviting them to the HF or through invitations by Village Health Teams (VHT). In one HF in a rural setting, formal “treatment supporters” assigned to these minors provided written informed consent on behalf of some caretakers who could not come to the health facility. Following consent/assent, an audio recorded interview that lasted between 45 min and 1 h was conducted in a language preferred by participants. The Principal Investigator (PI), a native speaker of Luganda and fluent in English together with a research assistant who is a native speaker of Rutooro and fluent in English conducted the interviews. Their vast experience working with youth in resource limited settings enabled them to easily connect with participants and establish a rapport that created a cooperative atmosphere in which participants freely shared their experiences. During each interview the PI or the research assistant observed and wrote some non-verbal characteristics of the participant such as physical appearance, demeanor, speech tone and aptitude to create a participant profile. None of the selected participants declined to take part but two became emotional and broke down during the interview sessions, which compelled us to stop although they expressed their ability to proceed to the end. We referred them to a peer counsellor who was always available at the HF.

### Ethics

Ethical approval was obtained from Uganda National Council of Science and Technology (UNCST), the Institutional Review Board (IRB) of The AIDS Support Organization (TASO) in Uganda, and the ethical committee of the Vrije Universiteit Brussels (VUB) in Belgium, reference number B.U.N. 143,201,835,870. We obtained written informed consent/assent from all participants and written informed consent from parents/caretakers of minors. The identities of participants are concealed in the findings and pseudonyms are used in some cases. At the end of the interview each participant was reimbursed with the equivalent of 7 USD to cover transportation costs.

### Data management and analysis

Data in audio files were transcribed verbatim by postgraduate students fluent in the concerned languages. Interviews conducted in local languages were translated to English and all transcripts were checked against the original audio recordings by the PI for accuracy. Data analysis was guided by the inductive thematic strategy [30]. Two authors, EK and SV, read the transcripts several times to get immersed in the data and independently coded 7 interview transcripts. The codes were then discussed, harmonized and collated into 6 potential main themes by a group of 5 authors EK, SV, DR, AE and JD in an iterative manner that involved returning to the data to understand and clarify the codes. The developed codebook was then used by EK to code all the remaining transcripts while remaining open to new codes, emerging sub-themes and main themes. Coding was done using the software NVIVO version 10 and all the data related to each potential main theme was gathered. The sub-themes and main themes were then checked in relation to the coded data extracts to ascertain their suitability in representing the data and covering its breadth. We finally named and defined the main theme, mapped their boundaries and established the relationships between sub-themes and main themes to complete the analysis (see Additional file 2: Table S2, for the main themes and sub-themes derived from the analysis). In the findings we presents results structured as themes of barriers and themes of facilitators.

### Findings

#### Characteristics of participants

Thirty five youth living with HIV/AIDS (16 females), aged 12–19 years (mean age = 16.2, SD = 2.37) took part in the study. Majority of them (88%) were perinatally infected. At the time of the study only two participants had never been to school while 13 were still in school. See Additional file 1: Table S1 for additional characteristics.

### Barriers

Participants experienced a number of barriers related to living with HIV/AIDS, which were clustered into Management of ART and illness; Fear, negative thoughts, and self-devaluation; Lack of meaningful and supportive relationships; Reactionary attitudes and behavior from others at school; and Financial challenges.

#### Management of ART and illnesses

Living with HIV requires lifelong treatment with ART and is associated with frequent opportunistic infections especially when optimal adherence to ART is not achieved. The ART medication however poses challenges and limitations within the social spheres of YLWHA. The youth voiced several barriers to attend school while at the same time following their strict medical regimens in addition to sporadic illnesses. Many stated that it requires a lot of courage to take medication daily at school especially due to fear of being identified as HIV positive and the discrimination that would follow. Those who were or had been in boarding schools noted that they had to take medicine in the dormitory or in the sickbay in full glare of others; something they were not comfortable with. One participant cited noisy pill bottles as alarm bells that alert everyone that someone is on medication for HIV, yet they need them for safety and potency of their medicine as advised by health workers.

“*The first problem is that we have these bottles for medicine. In boarding school, we have problems with these bottles because they make noise and other children make fun of us. Whenever they hear that noise from the bottles, they gather around to laugh and make fun of us. Even those who did not know about your status can now know”* (19-year-old school dropout male).

A 13 year old female participant, who had been on ART for about 1 year reported that her science teacher one day said to the class that HIV/AIDS has no cure. She wondered why she had to keep taking drugs that do not cure. Her resentment of medication led to deterioration of her health. This caused her to leave school for a period of 2 school terms. ART side effects was a prevalent theme cited by participants. Dizziness, general malaise, nausea and tinnitus were among the commonly reported side effects. These curtailed the ability of YLWHA to regularly attend class, pay attention and grasp what was being taught like their HIV-negative peers. Prisca, a 19-year-old female noted that she needs at least 30 min of sleeping after taking drugs prior to engaging in other activities. From their experiences, side effects were further aggravated by inadequate and poor meals at school. Akiiki, a 17-year-old male wondered how he could feed well (with fruits and vegetables) as advised by the doctor when schools cannot provide such foods.

Almost half of the participants reported that quite often they became sick due to infections such as cough, flue, skin rashes and malaria, to which they are more vulnerable than their peers. With these illnesses, they failed to regularly attend school and had to frequent health facilities for treatment and sometimes hospitalization. As a result, they lost school time, lagged behind other students and got frustrated with their poor performance and slow academic progress. They noted spending a considerable amount of time during the school term visiting ART clinics for their refills and management of intermittent illnesses as a 17-year-old male participant narrates in the quote below.

*“Yes, because the days for coming to the clinic would coincide with class time and this was not understood by the teachers. This led to punishment for absenteeism in school. I did not have any trusted person in school to disclose my situation to so that I may be officially known as one who goes for drugs during school times”* (17-year-old school dropout male).

These frequent illnesses were additionally reported by some participants as signs upon which others at school suspected them of having HIV and consequently isolating them. For some, the illnesses that started before school enrollment hindered or delayed their enrollment causing them to feel out of place when they sat in the same class with younger children. One 18-year-old, perinatally infected girl reported how these sporadic illnesses hindered her enrollment in school in the quote below.

*“I have never gone to school. When I was young and the time of going to school reached, I became sick. I would be sick one week and well another week and sick again the following week. My grandmother said this child will not manage school and so they did not take me there”* [18-year-old female, never been to school]

Although some participants reported side effects, fear of disclosure and subsequent discrimination associated with ART, all participants portrayed high regard for their medicine and remarked that their life depended on it if they adhered well. Therefore, they quickly decided not to participate in any activity that interfered with their timely routine medication. As a result, those who had not made proper plans to integrate treatment into their schooling and make appropriate maneuvers to overcome barriers reported above, did not enroll or studied with difficulties or were quick to drop out.

#### Fear, negative thoughts, and self-devaluation in YLWHA

The HIV diagnosis and subsequent disclosure of a seropositive status to YLWHA created a huge emotional burden to them. The youth carried this burden to school and school situations often aggravated it. Fear appeared as a common theme in all discourses of YLWHA. They feared death since they were aware that HIV/AIDS is a fatal incurable disease, and many had already lost a parent to it. They also commonly reported fear to disclose their status to others because they expected to be stigmatized, scorned, ridiculed, gossiped about, insulted, and isolated. Consequently, many kept their status secret at school but were in constant fear of unintended disclosure that would arise due to their health and medication as already discussed above and due to the non-confidentiality of those they trusted.

*“At school when you have HIV you don’t enjoy like other children because you know you have HIV and you fear that they will find out. So, you stay alone unless if you have a friend also with HIV in the same school”* (13-year-old female in a day school).

Those whose status was known in school did not want to make contributions in class for fear of being laughed at by fellow students in case their contributions were wrong. Some reported that they feared infecting others during normal interactions and games and be held responsible. The fear reportedly caused YLWHA to isolate themselves, stay solely with other HIV positive youth they knew and to miss out on the potential support that others would render them. Some even suggested having a school for themselves so that they do not have to worry about others knowing their status.

“Thinking death” was another emotional load that YLWHA revealed, especially those who had been on ART for less than 2 years. Other people typically did not help either, as they also carried a similar view about them. As a result, many participants reported that their HIV-negative peers at school used demoralizing statements such as ‘you are finished’, ‘you are a walking dead’, ‘you are a ghost’ and ‘the treatment for HIV is a spade and a hoe (death)’. These statements caused YLWHA to feel sad, worry all the time and to cease medicine. Some reported hating anything and any one that burdened them since they believed that their days were numbered. Those who did not have enough support and individual capacity to cope with such utterances, hated school and dropped out. The “thinking death” was also prominently reported in parents and caretakers of YLWHA causing them to discount YLWHA out of the future family plans. As a result, they denied them education and good material supports like clothes and food.

*“People are ignorant, like they think children with HIV can die any time, you know they call us ‘victims’. Like they can say ‘why do you spend money or school fees on someone who is going to die?’, as if for them they will not die”* (18-year-old school dropout female).

Many youth viewed themselves as worthless and similarly other people took the same stance of them. Some YLWHA believed that HIV preclude them from studying and progressing academically like other HIV-negative peers, and as a result, they refused to enroll or stay in school.

Most participants that dropped out of school also reported failure to grasp what was being taught at school and even suggested to their caretakers that they preferred to stay at home. For some, the decision to drop out of school came because they experienced slow academic progress due to their health as already reported.

#### Lack of meaningful and supportive relationships

All the youth we interviewed had lost one or both parents and as such, they reported missing parental care, love and advice which they deemed necessary in life. Those who had one parent and those who were living with caretakers additionally reported neglect, differential treatment and mistreatment. Many narrated situations where they were treated inferior to other children in the home, typically perpetuated by stepmothers who disvalued and mistreated them. This unfair treatment was exhibited in being denied scholastic materials. When in school, they mentioned situations where they had no one to visit them, encourage them, and to give them the necessary personal essentials.

*“I used to go to school, but I stopped in P3 because my step mother told my father that I was just wasting money and time to go to school since I was performing poorly”* (14-year-old school dropout male).

#### Reactionary attitudes and behavior from others at school

The youth reported several stressors that arose from interacting with other people at school. They had to contend with varied and divergent views, attitudes and behaviors about HIV/AIDS and those infected with it. Rumors and gossiping about them was variously reported. They described several instances where other people talked, pointed fingers, teased and made them a laughing stock. Some female participants reported being judged by others that they got HIV through prostitution or that they were cursed. Many participants reported these instances as being prevalent in schools

*“I said some are ignorant about HIV, also people think we are useless, we are going to die very soon, and some believe that we were cursed to have HIV at this age”* (19-year-old school dropout male).

Some youth noted cases where they had been discriminated by others at school and circumstances where they were considered weak and not fit to participate in activities like sports and games. One 19-year-old male narrated how a teacher stopped him from playing football because he was ‘sick’ [had HIV]. This caused him to feel excluded from the rest of the students. Three female participants further said that it affected them when others taunted them that they are sick or on treatment or have a virus. Such statements often excluded them from possibilities of participating in school activities.

*“Discriminating me is the worst thing that has really disturbed me. Like pin pointing at me every time and even telling me that I am sick continuously by teachers and students, yet I know that am sick”* (18-year-old female in a day school).

Isolation of YLWHA was normally done for fear that they would infect others. The youth reported that such fears occurred due to ignorance of others about ways in which HIV is transmitted. A few female participants reported that others isolated them because they did not trust them. According to these participants, other HIV-negative peers at school thought that they could deliberately transmit the virus to them. As a consequence of these attitudes and behaviors, many YLWHA concealed their status at school and isolated themselves from others, for fear of their status being known and being caused to feel different from the others. However, inadvertent status disclosure was reported to occur, especially in boarding schools due to living with many peers for a long time and due to the indiscretion of teachers, school nurses and matrons that some YLWHA would trust to disclose to. This breach of trust always left YLWHA muzzled and unhappy at school.

*“Me, I knew he [teacher] was going to tell other teachers and all the children at school would later get to know about me and we used to know that, that teacher does not keep secrets. He told a child at school who was related to him”* (14-year-old school dropout female).

The non-disclosure of status at school meant that YLWHA could not benefit from any supportive measures that the school community would put in place for them. Many reported challenges seeking permission to leave school as already reported under the main theme, management of ART and illnesses.

#### Financial challenges

All the youth we interviewed stated financial constraints because they had lost one or both parents who would fend for them. In several cases, they also reported being neglected by their parents and caretakers. They therefore lacked some fees that public schools levy for lunch, uniforms and books or tuition fees for those who had no access to a public school and had to go to a private one. As a result they were always sent away from school. Some few in boarding schools reported that they could not afford to supplement the school food and that they also lacked clothes. Majority of the participants stated that they lacked transport fares to go to ART clinics for refills and this affected their medication adherence, health and schooling.

*“I could not manage the tuition after losing my mother and now my grandmother could not afford to pay for me in school”* (17-year-old school dropout female).

*“My grandfather was the one who was paying for me from primary 1 to 4. He then had to hand over the responsibility to my father who said I should first hold on. I had to start working in the quarry to earn some money to pay for school fees, which I did up to primary 7. I could not complete the class because I failed to pay in the final term of primary 7”* (17-year-old school dropout male).

### Facilitators

Besides the barriers, participants also identified facilitators of their life quality, which were clustered into practical support at school, home and community; counselling, encouragement and spirituality; individual coping strategies; hopes, dreams and opportunities for the future.

#### Practical support at school, home and community

The youth who had selectively disclosed their status appreciated support from various stakeholders in the school community. Although it was a challenge identifying individuals to confide in, when such people were identified, they played a meaningful facilitative role for these youth in school. From school authorities, these youth received transport and school leave permits to attend their clinic appointments. Reminders and encouragements to adhere to medication were provided by some teachers. Teachers additionally provided remedial classes, offered special consideration in case of missed examinations and shared their food with YLWHA in some few cases. One participant reported that, family members assisted him to pick his medicine from the clinic while he was at school.

*“Also let us say I am in examinations, my sister picks for me drugs as well as my mother”* (18-year-old male in a day school).

Those who were or had been to boarding school suggested that receiving ART for the whole school term and reducing waiting time at the ART clinic would redeem time to keep up with school activities.

The youth mentioned several times that compassionate people came to their rescue and provided food and scholastic materials. These were notably friends at school who would share with them the little they had, school matrons who would share with them food and neighbors in the community who out of empathy would give them clothes, food and some money. They additionally appreciated a small financial contribution that was occasionally provided at the ART clinics to enable them meet transport fares from the clinic back to school and home.

Two participants reported having a part-time jobs at the ART clinics and in the community that they were engaged in during the weekend, ART clinic days and school holidays. With this small income, they were able to meet some of their financial needs at school such as scholastic materials, uniform and meals. Some youth also reported receiving support in form of school fees from individuals, community-based organizations and non-governmental organizations.

*“Yes, I used to pay my own school fees after carrying water for people but now I got a sponsor this year”* (18-year-old male in a day school).

#### Counselling, encouragement and spirituality

To lessen the emotional burdens, many YLWHA relied on counselling from health workers, teachers, family members and friends. This enabled them to accept their status and to start living positively with a future perspective. Counselling was based on facts that, people of all ages die, with treatment HIV can be managed, and that many people suffer from other terrible chronic diseases such as diabetes, hypertension and cancer.

*“I cried and asked myself how this had happened for me to get HIV, but the nurse counselled me and told me about other children who have HIV and I became strong”* (14-year-old male in a day school).

Testimonies of individuals who had lived with HIV for a considerable period were a basis to encourage the youth to be positive about their future. The youth also reported drawing courage from being able to perform better than HIV-negative youth in sports and academics at school. Additionally, many reported that they trusted God and prayed for good health, wisdom, courage, strength and cure in future. As a result, they were more optimistic that all will be well with them in future.

All school going participants reported receiving encouragement and advice from health workers to live positively within the school community by adhering to their treatment and coping with emotional challenges that would often arise at school. While some people were privy to the status of YLWHA by virtual of relationships with them such as caretakers/parents, health workers, counsellors and fellow YLWHA, the youth also voluntarily opted to disclose to some other people they deemed trustworthy at school. These were close friends, teachers, school matron and school nurse. They reported carrying out all the due diligence in deciding whom to disclose to in order to avoid rumors about them but instead obtain support and care

*“It helps [to disclose to someone] but how can you tell the teacher? At times you go and tell the teacher and which type of teacher do you tell? There are some teachers who talk and talk and talk what even some one has not said. Now that is bad. You must know the teacher you have to tell. And you study the teacher and make him/her your friend and then you tell him/her”* (19-year-old male in a boarding school).

One youth reported being part of a supportive network in which he was empowered to face challenging social settings such as schools. In this peer support network, they shared experiences of living with HIV and encouraged each other.

*“It is courage, advice from people and friends who have the same problem [HIV] and who had ever been in boarding. They gave me some tips when I was coming to boarding”* (16-year-old male in a boarding school).

#### Individual coping strategies

Some participants reported keeping medicine with the school nurse as helpful and noted that prior arrangements to have their medicine could be made on the days and time the nurse would not be around to attend to them. A few who chose to keep their medicine in the dormitory stated that it was always under lock and key in their suitcases and that they only accessed it in absence of others or in hiding within their beds as typified in the quote below.

*“I remove my drugs from the suitcase when I am going to dress up, then I put it under my pillow and I cover it under my bed and when I come back from class, I just open the pillow, take my drugs and I go back”* (18-year-old female in a boarding school).

School going youth mainly chose day schools because it appeared easier to juggle schooling, medication and frequent visits to ART clinics.

Almost all youth reported that keeping secrecy of their status, staying with fellow HIV-positive youth and prevention of suspicions about their status can help to survive in school. They noted that the school provides several options for them to make friends and interact in order to curtail their worries and fears. Most participants upheld games and sports at school as vital distractions that calmed their wandering minds.

*“To keep at school, you don’t get a lot of thoughts, at school you are happy, you study, you play so you keep happy the whole day”* (16-year-old female in a day school).

In a few cases, participants reported changing school whenever their status was known. According to them, they would enter a new social environment in which they would be considered like others and thus avoid discrimination. While this was a failure for schools to integrate them, it seemed an active coping strategy for YLWHA to continue schooling.

#### Hopes, dreams and opportunities for the future

All the youth viewed the school as a crucial resource to facilitate their personal development and strengthen their social position and self-worth. Those who were still in school upheld the importance of schooling, stating that through school they could attain their hopes and dreams. Many stated that they wanted to be very important professionals in the future and provide solutions to societal problems. For instance, Christine, a 14-year-old girl stated that she wanted to study and become a nurse to treat children living with HIV/AIDS. All participants noted that education could guarantee them a better future through better opportunities for employment and skills for independent living. A few school going participants reported being motivated at school whenever they excelled in their studies and received praises from teachers and their peers. They also reported getting individually empowered when they attended conferences in which they shared experiences and learnt to express themselves in a group. This was noted to boost their self-esteem at school.

## Discussion

In this qualitative study, we investigated, via semi-structured interviews with 35 youth living with HIV/AIDS (YLWHA), HIV-related barriers and facilitators to attend school in Uganda as perceived and experienced by YLWHA.

Our findings revealed a significant burden to adhere to the highly treasured ART regimens while simultaneously maintaining secrecy of the serostatus that YLWHA grappled with. This seems to be uniquely so in school communities where they live and interact closely with people with divergent views on HV/AIDS, and people living with it. YLWHA always had to weigh the risk of taking ART at school and the benefit of schooling. Since ART showed more immediate outcomes of improved health compared to schooling, they were always quick to disregard the latter. The key risks associated with taking ART at school as reported by participants were unintentional disclosure and subsequent discrimination. Although HIV/AIDS has been theorized as a concealable stigmatized identity [31, 32], our findings show that within the school context YLWHA are highly noticeable. Both internalized and externalized overt and covert forms of HIV-related stigma were reported in this study. Our study reinforces findings from several other studies on disclosure and medication adherence in YLWHA, reporting HIV-related stigma as a key hinderance in school [6, 24, 26, 33, 34].

In this study, most of the YLWHA chose day schools because it appeared easier to juggle schooling, medication and frequent visits to ART clinics than in a boarding school. Although they would take medicine at home, their status was always involuntarily revealed at school by those who met them at ART clinics. Also, some teachers they trusted to disclose to, and other people in school who had prior knowledge about their status in the community often did not meet their expectations of confidentiality.

Studies have revealed that absolute concealment of HIV-status in school is barely possible [6, 24] although YLWHA attempt it for fear of HIV-related stigma, given that school communities have not been adequately prepared to integrate them. Our findings relate to cases where YLWHA felt out of place and believed that schooling was not meant for them. Although some reported measures to cope such as preventing suspicions by hiding medicine, self-isolation and partial disclosure to very few trusted people in school as similarly reported by Mutumba and colleagues [26], such strategies are not sustainable. These youth are often involuntarily disclosed due to congestion, lack of privacy and rumors in the school community. We envision a supportive school community as one in which not only the YLWHA are prepared to overcome HIV-related stigma but one in which all the students are sensitized regularly to see YLWHA as fellow students but with special needs. To achieve this, causes and effects of HIV-related stigma should be addressed by teachers bearing in mind the fragility of YLWHA. Such well-designed sensitization campaigns can dispel misconceptions and change the attitudes and beliefs about HIV hence transforming the school community from a stigmatizing one to a supportive one. Our findings show that YLWHA welcomed various forms of support ranging from counselling to material provisions that were offered to them at school as similarly found by other researchers [24, 34–36]. This kind of support however, benefited a few YLWHA who took a risk to partially disclose to some trusted people. In order to scale up support, a favorable social environment needs to be created at school level where YLWHA can freely choose to fully or partially disclose their status without worrying about the negative reaction or other baleful consequences. This requires continuous sensitization and education programmes in schools to address HIV-related stigma.

The ramifications of stigma within school should not be taken lightly as it has adverse effects on the wellbeing of YLWHA, including medication adherence, emotional well-being, social inclusion, and school engagement. HIV-related stigma and its implications on schooling have been widely studied in resource limited setting [37–39]. In this study, the youth reported dropping out of school, changing school, isolating themselves and refusing to participate in class activities whenever their status was involuntarily disclosed to their peers due to anticipated and enacted stigma [40]. Supportive school environments should go beyond accepting YLWHA and trying to integrate them into their existing system. Instead, they should work towards creating realistic possibilities for them to be active participants in school. Although HIV education is part of the education curriculum in Uganda as a crosscutting theme, what is discussed especially about HIV-related stigma is not clear. The Uganda Demographic and Health Survey found that less than half of young people had comprehensive knowledge about HIV/AIDS [41]. Much emphasis in HIV/AIDS education is devoted to prevention strategies [42] as these are deemed appropriate for youth who are not or are getting sexually active. It is also a concern whether teachers are satisfactory trained and have the competencies to deliver this message to students. Our results allude to deficiencies in teachers’ knowledge and skills during delivery of information about HIV/AIDS. Participants faulted them of further stigmatizing them when they used them as examples in class, and when they spoke negatively about HIV/AIDS, using statements that caused YLWHA to lose hope. A study conducted in Thailand also found that teachers lacked knowledge on HIV/AIDS [20]. It is therefore imperative for interventions to start with training teachers and other school staff on current facts about HIV/AIDS and appropriate methods of transmitting this knowledge to school youth and parents/caretakers since teachers are in a privileged position of interacting with these two groups.

Our findings also showed that many YLWHA had lost one or both parents as is often the case with perinatally infected youth [34, 35]. As a result, these youth lacked appropriate parental guidance, love and care that would support them in a stressful school environment. This devoid of support needs to be filled up by other people in the social spheres of YLWHA and since these youth spend more time at school and they reported mistreatment and neglect at home, teachers need to reflect on their potential to take up this role as auxiliary caretakers. In a related study with Peer Educators conducted in the same area, participants suggested that each YLWHA would be assigned to a special teacher they called a “guardian teacher”. Such a teacher would preferably be HIV-positive and would cater for the psychosocial needs of the youth assigned to him/her at school. Although this seems plausible, Ugandan schools are highly populated and teachers’ workloads are significantly high [43] but also, there are other students with special needs that require due attention from teachers. We therefore stress self-empowerment of YLWHA coupled with transformation of the entire school community into a supportive community for all vulnerable students. With regard to self-empowerment of YLWHA, we found that supportive organizations and peer groups in the community that gave YLWHA a platform to share experiences and devise solutions were instrumental in preparing them to confront the school environs. The involvement of all students in clubs, games and sports is additionally recommended since it provides distraction, skills and social integration for all students as reported in this study and others [26, 35]. Such integration however is not accidental and cannot be coerced but takes the form of protracted efforts to understand the needs of vulnerable students [44]. Because youth at school are a captive audience and are highly receptive to information [44], curricular and extracurricular measures at school are necessary to drive integration of YLWHA by addressing all the misconceptions, negative beliefs and attitudes about HIV and overtime a spillover effect would be realized in communities outside school as changed youth take the messages back to their communities.

We found that all YLWHA had financial constraints to attend school, which has been similarly reported in several studies with YLWHA e.g., [6, 34, 45, 46]. Also, the perception of these youth as useless, weak and unworthy of any form of education or employment meant that they were often sent away from school or dropped out. The lack of education leaves them in a viscous cycle of poverty since it limits their prospects of employment in future [47]. HIV/AIDS and poverty have been widely studied in Sub-Saharan Africa [48, 49] but in adult populations. The recommendations and interventions available do not apply to the current and future financial needs of YLWHA who at times do not have adults to care for them. In this study, the youth strongly voiced the impeccable value of education and its potential to elevate their economic status. Several international agencies attest to this correlation and have led to the promotion of education. However much the Ugandan government provides free access to schooling, public schools are not accessible to all youth since they are few and usually distant from their communities. These schools also levy some fees for feeding, uniform and scholastic materials which may be deterrent to most YLWHA. As a way of providing more support for YLWHA, we recommend fee waivers in all schools that YLWHA would wish to attend. Additionally, these youth can receive scholastic materials at the ART clinic along with their medication. This would require a policy shift and close collaboration between the line ministries of health and education.

Lastly, we posit that schools are in a more privileged position to galvanize support around YLWHA. In addition to their internal supportive role, school staff could stretch their mandate beyond school boundaries and take their educative role to the larger community. This could involve collaboration with caretakers, health professionals, and other significant stakeholders to create supportive networks around YLWHA that would dispel negative attitudes about HIV/AIDS and YLWHA in addition to providing support that can cater to the different QoL domains for YLWHA in schools.

### Key recommendations

- Teachers should provide regular sensitization to all students to dispel myths and misconceptions about HIV/AIDS in order to fight HIV-related stigma.
- All school staff need to be trained on the current facts about HIV/AIDS and appropriate methods of transmitting this knowledge to school youth.
- Youth living with HIV/AIDS should be empowered by significant people in their lives, such as teachers, health workers and caretakers to overcome stigma and cope with HIV/AIDS in school.
- Schools should promote extracurricular activities such as games and sports for all learners to build social cohesion, physical fitness and to tame the wandering minds of YLWHA.
- School authorities should provide fee waivers and scholastic materials to YLWHA in order to encourage them to attend school.

### Study strength and limitations

Although we used a small purposively selected sample of participants, our findings, revealed pertinent issues that can be investigated further in large scale multimethod studies. With this sample we were able to get into depth of barriers and facilitators for YLWHA to attend school which provides a clear understanding of the school environment for YLWHA and how supportive school environments can further be promoted. Our findings were based on experiences and perceptions of YLWHA which are prone to recall bias and personal preferred reporting even with the appropriate probes. We contend that the school staff, and caretakers at home would have been involved to provide more information to corroborate or counter the views of youth in order to arrive at more substantiated findings.

## Conclusions

This study unearthed unique barriers and facilitators for YLWHA to attend school. Most of the barriers arose due to HIV-related stigma and financial challenges whose genesis transcends school borders. While YLWHA reported measures to cope, and support from other people, these were non-sustainable and on a limited scale due to disclosure apprehensiveness at school and the indiscretion of those who learn about their status. To promote supportive school environments for YLWHA, integrated curricular and extracurricular interventions are necessary to promote comprehensive HIV knowledge, dispel misconceptions about HIV and consequently transform the school community from a stigmatizing one to a supportive one. Overtime the entire community gets transformed as students carry the messages back to the community. Such measures require an active role of school staff who would need prior in-service training to understand what is developmentally and culturally appropriate and to engage with other stakeholders outside school to create supportive networks around YLWHA.

## Supplementary information

**Additional file 1: Table S1.** Characteristics of study participants (*n* = 35)

**Additional file 2: Table S2**. Themes and main themes derived from thematic analysis

**Additional file 3.** Information & informed consent

## Data Availability

The dataset generated and analyzed during the current study is not publicly available because we did not seek consent from participants to share the data publicly. However, this dataset is available from the corresponding author on reasonable request.

## Abbreviations

ART: Antiretroviral Therapy
DHO: District Health Office
HF: Health Facility
HIV: Human Immunodeficiency Virus
IRB: Institutional Review Board
PI: Principal Investigator
QoL: Quality of Life
SSA: Sub-Saharan Africa
TASO: The AIDS Support Organization
UNCST: Uganda National Council of Science and Technology
UPE: Universal Primary Education
USE: Universal Secondary Education
VHT: Village Health Team
VUB: Vrije Universiteit Brussels
