# Supplementary material for: Experiences and perceptions of youth living with HIV in Western Uganda on school attendance: barriers and facilitators
Source: BMC Public Health. 2020 Jan 17;20:79. doi: 10.1186/s12889-020-8198-7 (PMC6969460; doi:10.1186/s12889-020-8198-7)
Supplement: Supplementary file 1 — Additional file 1: Table S1. Characteristics of study participants (n = 35) [file 12889_2020_8198_MOESM1_ESM.docx]

| **Characteristic** | **Frequency (percentage)** |
| --- | --- |
| **Age (years)**  Mean (SD) | 16.2 (2.37) |
| **Sex**  Males  Females | 19 (54)  16 (46) |
| **Source of HIV infection**  Perinatal  Rape  Unknown | 31 (88)  1 (3)  3 (9) |
| **Schooling status by time of interview**  In school  Dropped out of school  Never gone to school | 13 (37)  20 (57)  2 (6) |

***Table 1: Characteristics of study participants (n=35)***
