# Supplementary material for: Experiences and perceptions of youth living with HIV in Western Uganda on school attendance: barriers and facilitators
Source: BMC Public Health. 2020 Jan 17;20:79. doi: 10.1186/s12889-020-8198-7 (PMC6969460; doi:10.1186/s12889-020-8198-7)
Supplement: Supplementary file 2 — Additional file 2: Table S2. Themes and main themes derived from thematic analysis [file 12889_2020_8198_MOESM2_ESM.docx]

| **Main themes** | **Codes/themes** |
| --- | --- |
| **Barriers** |  |
| Management of ART and illnesses | Frequent illnesses  Drug side effects  HIV has no cure  Keeping medication in dormitory  Daily medication  Value of treatment  Keeping medicine at school  School food |
| Fear, Negative thoughts, and self-devaluation in YLWHA | Worries / Sadness  Thinking about death  Isolation  Suicidal tendencies  Fear of (disclosure, death, infecting others)  Self-image of worthlessness /self-hate  Association of status with death  Loneliness  Sad memories of parental loss  Questions of why they are the ones with HIV |
| Lack of meaningful and supportive relationships | Lack of parental care  Parental / caretaker neglect  Loss of friendships  Non-confidentiality of school staff |
| Reactionary attitudes and behavior from others at school | Discrimination  Abuse / Insults  Mistreatment  Rumors/gossiping in the school  Bullying at school  Isolation  Ignorance  Judging YLWHA  HIV as a curse  Considered weak/unfit  YLWHA distrusted by others  Laughing at YLWHA  Fear of infection  Unintended disclosure by others  Constant reminders about their status |
| Financial challenges | Transport fares to clinic  School fees  Clothes  Feeding  Paid job |
| **Facilitators** |  |
| Practical support at school, home and community | School nurse  Transport to ART clinic  School leave permits  Assistance to pick drugs from ART clinic  Partial disclosure  Sensitization of others  Peer support |
| Counselling, encouragement and spirituality | Information about medication  Testimonies of others living with HIV  Counselling  Trust in God (spirituality)  Acceptance of status  Encouragement and advice to (adhere to treatment, cope with emotional difficulties)  Learn how to live with HIV in a certain setting e.g. boarding school |
| Individual coping strategies | Installing a daily routine  Attending a day school  Hiding medicine in suitcase  Hiding when taking medicine  Treatment planning  Use of alarm clock as reminder  Secrecy/preventing suspicions  Distractive activities  Interacting with fellow YLWHA  Changing school  Non-disclosure of YLWHA |
| Hopes, dreams and opportunities for the future | Hopes and aspirations  Developing skills  Positive contribution to the community and society  Hopes and dreams  Chances of getting a job  Opportunities for development  Living independently  Academic achievements |
|  |  |

***Table 2: Themes and main themes derived from thematic analysis***
