# Supplementary material for: Experiences and perceptions of youth living with HIV in Western Uganda on school attendance: barriers and facilitators
Source: BMC Public Health. 2020 Jan 17;20:79. doi: 10.1186/s12889-020-8198-7 (PMC6969460; doi:10.1186/s12889-020-8198-7)
Supplement: Supplementary file 3 — Additional file 3. Information & informed consent [file 12889_2020_8198_MOESM3_ESM.docx]

# Information & informed consent

The interviewer starts with:

a) Introducing him or herself (name, profession, affiliation, …)

b) Explaining the objectives of the research

c) Outlining the method and course of the interview

d) Discussing in detail the ethical protocol and signing the informed consent form

- Confidentially: names won’t be used, person’s won’t be able to be identified and information won’t be shared with others
- Unconditionally: (non-)participation doesn’t bring along consequences, termination of participation is allowed at any time without baleful outcomes
- Informed consent: declaration of understanding and agreement with this information and giving permission to use this information for research
- Psychosocial support: possibility to be linked up with professional caregivers for psychosocial support

e) Practical information:

- Possibility to talk in own language through a translator
- Permission to record the interview on tape

f) Asking for eventual remaining questions on this information

# Setting the scene / Introduction

- Can you tell me about yourself, about who you are?
- What things in your life make you (un)happy these days?
- Can you describe a typical day in your life?
- Which days are different and why?
- Who do you live together with?
- (Where) do you meet up with friends?
- Do you go to school? (which school and which class are you in?)
  - If yes, do you like schooling? What do you (dis)like about it?
  - If not, how come you don’t go to school? Have you been to school before?
- Do you receive any support / services?

# Experiences of living with HIV/AIDS

- How did you get to know about your status?
- When and where did it happen? How long do you know about it?
- Did you discover it yourself or did someone else inform you about it?
- How did you react when you got to know that you are HIV positive?

a) Behaviour: what did you do?

b) Cognition: what did you think?

c) Emotion: what did you feel?

- Do you still have such thoughts/feelings/behaviours?
- If they changed, have they become better (more positive) or worse (more negative)?
- If they changed, what has caused the change/how did you overcome such feelings?
- If you were to summarize how you feel about living with HIV/AIDS nowadays, what word (emotion) would you use to describe it? Can you explain?
- Has HIV/AIDS had an impact on your life and your wellbeing? Did it bring any good and/or bad things in your life?
- What do you perceive as the greatest impact of HIV/AIDS on your life?
- On which life domains do you experience the largest effects (e.g. personal development, emotional wellbeing, physical wellbeing, interpersonal relationships, material wellbeing, social inclusion, rights, self-determination)?
- (How) Has living with HIV affected you
  - At home ?
  - At school ?
  - In the community?
  - Elsewhere?
- What should other people know about youngsters who live with HIV/AIDS? According to you, what is quintessential to know if people want to interact with and support them?
- What is the community attitude towards young people with HIV/AIDS?

# Disclosure

- Who else knows about your status? How did they react when they got to know?
- Did you tell them or did they find out?
- If you told them, how did you approach this? Was it easy/difficult for you?
- Was their reaction like you had expected?
- Would you wish to tell another person about your status?

1. At home
2. In your community
3. At school

- If not, what withholds you?
  - Are there any risks/consequences involved in disclosing to others?
- If yes, why do you want to do so?
  - Are there any benefits of disclosing to someone else at school or in the community?
- What would their reaction be if you tell them you are positive? Why?

# Perceived resources and barriers for living with HIV/AIDS in the school community

- How is it like to live with HIV/AIDS in your family / community?
- What sort of ideas live in your family and community on HIV/AIDS issues?
- What kind of an environment does this community offer to YPLWHA?
- What are the things that you find most support/strength in?
- What are the things that you find most challenging, that undermine your wellbeing ?
- What kind of an environment does the school offer to YPLWHA?
- Do you think that the school community is a good and supportive environment for youths living with HIV/AIDS?
- Why (not)?
- If you go to school:
  - What have been good experiences for you?
  - What helps you to live with HIV/AIDS in the school community?
  - What have been bad experiences for you?
  - What makes it difficult to live with HIV/AIDS in the school community?
- If you have never been to school:
  - Are there any barriers you experience that discourage you from going to school with HIV/AIDS?
  - Have you had or heard of any negative experiences within the school community that you relate to HIV/AIDS?
  - What should change to allow you to go to school?
- If you have been to school before (case of drop-out):
  - Have you had good experiences in the school community?
  - What helped you to live with HIV/AIDS in the school community?
  - Have you had or heard of any negative experiences within the school community that you relate to HIV/AIDS?
  - What is it that has caused your drop-out? It is related to your HIV/AIDS status?
  - What makes it difficult/impossible to live in the school community when having HIV/AIDS?
  - Would you like to return?
- If yes, what should change to make that possible?
- If no, what should change to make you want to return?

# Resilient school communities

- What good practices do you experience or see at home, school and community that make your life with HIV/AIDS better?
- What would you like to see changed in your environment? If you look at how things are handled in your school and community, what new recommendations would you like to make?

1. At home
2. At school
3. In the community

- What do you think can be done to improve your wellbeing and that of other young people living with HIV/AIDS?

1. At home
2. At school
3. In the community

- What could better facilitate school/community participation of yourself and other young people living with HIV/AIDS?

1. At home
2. At school
3. In the community

# Concluding questions

- Is there something you want to add to this interview, something we didn’t talk about but that you feel is important to you and want to share?
- Are there any questions you have for the researcher?
- Can we contact you later for further questions?

***Thank you very much for your time and response.***
